# Identified Seaweed Compound Diphenylmethane Serves as an Efflux Pump Inhibitor in Drug-Resistant *Escherichia coli*

**DOI:** 10.3390/antibiotics10111378

**Published:** 2021-11-10

**Authors:** Wen-Jung Lu, Pang-Hung Hsu, Chun-Ju Chang, Cheng-Kuan Su, Yan-Jyun Huang, Hsuan-Ju Lin, Margaret Lai, Gui-Xia Ooi, Jing-Yi Dai, Hong-Ting Victor Lin

**Affiliations:** 1 Department of Food Science, National Taiwan Ocean University, No. 2, Pei-Ning Road, Keelung 202, Taiwan; miss350100@gmail.com (W.-J.L.); chunju@mail.ntou.edu.tw (C.-J.C.); zxc214863@gmail.com (Y.-J.H.); angel810801@gmail.com (H.-J.L.); momoonelai@gmail.com (M.L.); guixia95@gmail.com (G.-X.O.); k921678@gmail.com (J.-Y.D.); 2 Department of Bioscience and Biotechnology, National Taiwan Ocean University, No. 2, Pei-Ning Road, Keelung 202, Taiwan; phsu@ntou.edu.tw; 3 Center of Excellence for the Oceans, National Taiwan Ocean University, No. 2, Pei-Ning Road, Keelung 202, Taiwan; 4 Department of Chemistry, National Chung Hsing University, 145 Xingda Road, South District, Taichung City 402, Taiwan; cksu@nchu.edu.tw

**Keywords:** seaweeds, efflux pump inhibitors, multidrug resistance, drug transporters, diphenylmethane, aromatic compounds

## Abstract

Drug efflux pumps are one of the major elements used by antibiotic-resistant bacteria. Efflux pump inhibitors (EPIs) are potential therapeutic agents for adjunctive therapy, which can restore the activity of antibiotics that are no longer effective against pathogens. This study evaluated the seaweed compound diphenylmethane (DPM) for its EPI activity. The IC_50_ and modulation results showed that DPM has no antibacterial activity but can potentiate the activity of antibiotics against drug-resistant *E. coli*. Time-kill studies reported that a combination of DPM and erythromycin exhibited greater inhibitory activity against drug-resistant *Escherichia coli*. Dye accumulation and dye efflux studies using Hoechst 33342 and ethidium bromide showed that the addition of DPM significantly increased dye accumulation and reduced dye efflux in drug-resistant *E. coli*, suggesting its interference with dye translocation by an efflux pump. Using MALDI-TOF, it was observed that the addition of DPM could continuously reduce antibiotic efflux in drug-resistant *E. coli*. Additionally, DPM did not seem to damage the *E. coli* membranes, and the cell toxicity test showed that it features mild human-cell toxicity. In conclusion, these findings showed that DPM could serve as a potential EPI for drug-resistant *E. coli*.

## 1. Introduction

Drug resistance has significantly increased uncertainty over treatment and medical costs, as well as the mortality rate from bacterial infections. It is estimated that by 2050, drug-resistant diseases could cause ten million deaths each year if no action is taken [1]. Attempts by pharmaceutical companies to reverse this trend have achieved limited success because of the slow development and release of novel antimicrobial agents [2]. Efflux pumps in bacteria are major contributors to drug resistance; bacteria can use transport proteins on the cell membrane to remove antibiotics that enter the bacteria. This, in turn, lowers the antibiotic concentration in the cell, thereby reducing antibiotic efficacy and increasing the bacterial survival rate. For example, the AcrAB-TolC transporter in gram-negative bacteria *Escherichia coli* can transport various drugs, such as macrolides [3,4], tetracyclines [5], fluoroquinolones [6], and β-lactams [7], across the inner and outer membranes [8,9]. Recently, the use of efflux pump inhibitors (EPI) has rapidly drawn attention as a promising approach to treat infections caused by pathogens expressing multidrug-resistant efflux pumps [10,11]. Their use in adjunctive therapy may enhance/restore the activities of existing antibiotics by interfering with efflux pumps; it may even allow therapeutically ineffective antibiotics to be re-introduced into clinical practice. EPIs could interfere with the function of efflux pumps by (i) functioning as a competitive/non-competitive inhibitor for drug efflux, (ii) interfering with efflux pump assembly or expression, and (iii) interfering with the energy source for active drug transport [12]. Several well-known effective EPIs, such as verapamil [13,14], reserpine [15], phenylalanine-arginine β-naphthylamide (PAβN) [16], and carbonyl cyanide *m*-chlorophenylhydrazone (CCCP) [17], have seen limited clinical use and development because of their cellular toxicity [11]. With the current shortcomings in EPI development, EPIs of natural origin have been drawing much attention. Plants are natural and sustainable; they are a promising source of potential natural EPIs due to their diverse phytocompounds and low toxicity [18]. For example, plant alkaloid piperine from black pepper (*Piper nigrum*) and long pepper (*Piper longum*) was found to potentiate the activity of ciprofloxacin against *S. aureus* strains expressing the drug efflux pumps MdeA and NorA [19,20]. Additionally, piperine could increase the bactericidal activity of rifampicin and significantly extend its post-antibiotic effect, by inhibiting drug efflux pump Rv1258c in *Mycobacterium tuberculosis* [21].

There has been a growing interest in seaweed bioactive compounds due to their availability, diversity, and productivity [22]. Seaweeds are rich in polysaccharides, pigments, fatty acids, polyphenols, and peptides, which have been proven to possess various beneficial biological activities, such as antioxidant [23], antimicrobial [24], anti-inflammation [25,26], anti-diabetic [27,28], and anti-cancer activities [29]. However, the studies evaluating the potential of seaweeds for EPI activity are limited. In a previous study, red seaweed *Gracilaria* sp. extract was found to potentiate the activities of drugs against *E. coli* overexpressing efflux pump AcrB [30]. In this study, an organic compound, diphenylmethane (DPM), was identified in the extracts of *Gracilaria* sp., and its EPI activity was investigated.

## 2. Results and Discussion

### 2.1. DPM Modulates the Activities of Macrolides and Fluoroquinolones against Drug-Resistant E. coli

In a previous study, the alcoholic extracts of red seaweed *Gracilaria* sp. were found to perform inhibitory activities against *E. coli* overexpressing the efflux pump AcrB; therefore, the alcoholic extracts of *Gracilaria* were analyzed using gas chromatography-mass spectrometry (GC-MS) to identify the potential compounds that inhibit efflux pump activities. The mass spectra of alcoholic extracts were deconvoluted using the AMDIS^®^ NIST Mass Spectral library, and the identification was accomplished by comparing the similarity of mass spectral fragmentation pattern and retention time with known reference molecules. As indicated in Supplementary Figure S1, the mass spectrum of the analyte identified from the alcoholic extracts shared a similar mass fragmentation pattern with DPM. As a result, DPM was purchased commercially and evaluated for its EPI activity against *E. coli* efflux pump AcrB.

The antibiotics clarithromycin, erythromycin, and ciprofloxacin have been reported as substrates of the *E. coli* efflux pump AcrB [31,32], and the construct *E. coli* Kam3 harboring pSYC-*acrB* (Kam3-AcrB) was used in the modulation assays. Our preliminary data indicated that DPM did not show any antibacterial activity at the highest concentration of 1000 µg/mL against Kam3-AcrB (data not shown). As shown in Table 1, the IC_50_ of clarithromycin, erythromycin, and ciprofloxacin were determined to be 175, 125, and 0.06 µg/mL, respectively. DPM at 7.81, 125, and 7.81 µg/mL could reduce the IC_50_ of clarithromycin, erythromycin, and ciprofloxacin by two-fold, respectively. In the modulation data, DPM at various concentrations could exhibit modulation activities for different antibiotics. A kaempferol glycoside kaempferol-3-O-β-d-(6”-E-p-coumaroyl) glucopyranoside (tiliroside) was evaluated for its modulation activity for various quinolone antibiotics and antiseptics against *Staphylococcus aureus* expressing the efflux pump NorA, and the data indicated that tiliroside at various concentrations exhibited modulation activities for different drugs [33]. Hossain et al. [34] conducted checkerboard assays for plant-derived isoflavone epicatechin gallate in the presence of various antibiotics against *E. coli*, with various FICI values ranging from 0.516 to 1.004. These results indicated that potentiating the activity of EPIs for various antibiotics could vary significantly.

### 2.2. Effect of DPM on Time-Kill Curves

Time-kill studies were used to observe the changes in the actual cell counts of bacteria with exposure to erythromycin in a given period [35].

**Figure 1:**
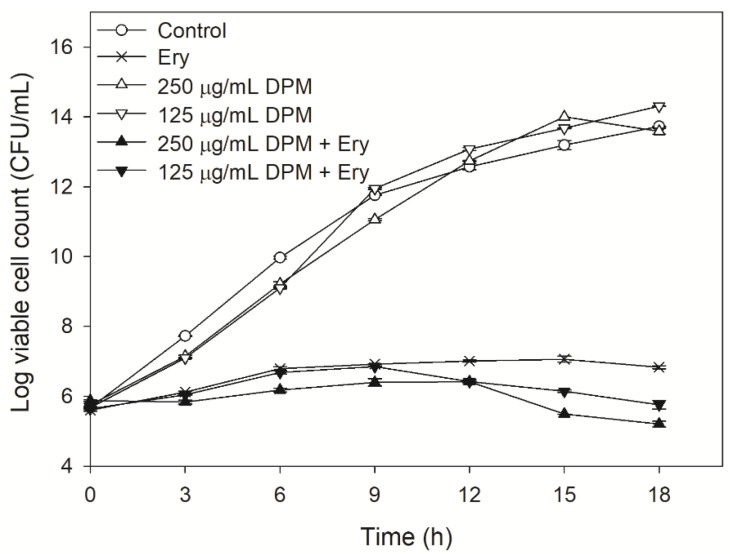
Time-kill curve of *E. coli* Kam3-AcrB with erythromycin, diphenylmethane, and in combination. Ery, Erythromycin; DPM, diphenylmethane. Data are expressed as mean ± SD (*n* = 3).

As indicated in Figure 1, the *E. coli* Kam3-AcrB (control), the Kam3-AcrB incubated with 250 µg/mL DPM, and the Kam3-AcrB incubated with 125 µg/mL DPM showed similar cell growth, indicating that DPM did not demonstrate antibacterial activity against Kam3-AcrB, which was consistent with the IC_50_ data. The addition of erythromycin to the Kam3-AcrB culture significantly reduced bacterial growth, with a cell count of 6.82 Log CFU/mL at 18 h. The addition of erythromycin + 125 µg/mL DPM to Kam3-AcrB culture exhibited cell growth similar to the erythromycin group at 9 h and gradually reduced the cell count to 5.7 Log CFU/mL at 18 h. The addition of erythromycin + 250 µg/mL DPM to the Kam3-AcrB culture exhibited the highest inhibitory effect on the bacteria, with a cell count of 5.2 Log CFU/mL at 18 h. Bacteriostatic antibiotic-erythromycin could be bactericidal in some instances, such as at high concentrations. A significant decrease in cell count was observed with the addition of 250 µg/mL and 125 µg/mL of DPM after 9 h and 12 h. Our data indicated that the combined use of erythromycin and DPM exhibited a higher inhibitory effect on Kam3-AcrB compared with the erythromycin group, with a reduction in cell count at 18 h by ≥1 Log CFU/mL.

### 2.3. DPM Reduced Dye Accumulation in E. coli Kam3-AcrB

**Figure 2:**
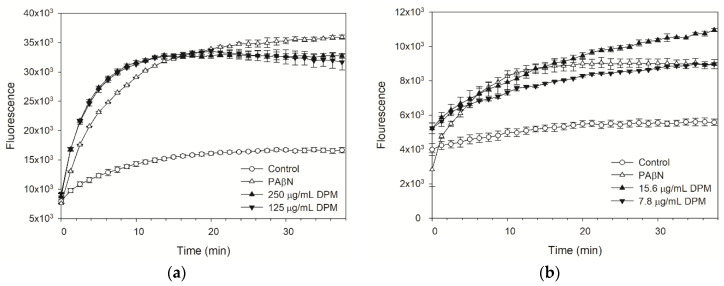
(**a**) H33342 and (**b**) EtBr accumulation of diphenylmethane in *E. coli* Kam3-AcrB. Data are expressed as mean ± SD (*n* = 3).

The dyes H33342 and EtBr were used to monitor the accumulated fluorescence in *E. coli* Kam3-AcrB. The RND (Resistance-Nodulation-Division) pump inhibitor PAβN was used as the positive control [36]. As indicated in Figure 2a, DPM at both 250 µg/mL and 125 µg/mL could increase the accumulation of H33342 in Kam3-AcrB, thus providing indirect evidence that DPM interferes with the efflux of H33342 by Kam3-AcrB. Similar results are indicated in Figure 2b, which demonstrates that DPM at 15.6 µg/mL and 7.8 µg/mL could dose-dependently increase the EtBr accumulation in Kam3-AcrB. Mouwakeh et al. [37] evaluated the phytochemical components of black cumin (*Nigella sativa*), such as thymoquinone, carvacrol, and p-cymene, for efflux pump inhibition activity and showed that all three compounds could increase EtBr (Ethidium bromide) fluorescence accumulation in methicillin-resistant *S. aureus*, thus demonstrating their application as bacterial-resistance modifiers. The dye accumulation data indicated that DPM possesses the potential to act as an EPI against *E. coli* Kam3-AcrB.

### 2.4. DPM Reduced Dye Efflux by E. coli Kam3-AcrB

**Figure 3:**
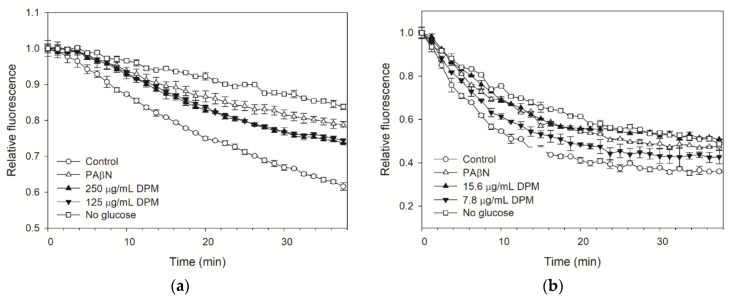
The (**a**) H33342 and (**b**) EtBr efflux assay with diphenylmethane in *E. coli* Kam3-AcrB. Dye was added to the drug-resistant *E. coli* cells, which were energized with glucose in the presence of DPM. H33342 (3 µM), EtBr (3 µM), glucose (25 mM), PAβN (20 µg/mL), and DPM. Data are expressed as mean ± SD (*n* = 3).

A dye efflux assay can be used to evaluate the activity of EPIs, providing direct evidence of whether the pump efflux has been interfered with in the presence of EPI [38]. To further evaluate the potential of DPM as an EPI against *E. coli* Kam3-AcrB, H33342 and EtBr efflux in Kam3-AcrB in the presence of DPM was monitored. As indicated in Figure 3a, Kam3-AcrB (control) could reduce H33342 fluorescence to 0.61 in 38 min, indicating the efflux of H33342 from the cells. The Kam3-AcrB without additional glucose energization featured an H33342 fluorescence of 0.84 in 38 min, which was higher than the control group. The addition of PAβN and DPM (250 µg/mL and 125 µg/mL) could slow the decrease in H33342 fluorescence, suggesting that these compounds could interfere with the H33342 efflux from the cells. Similar results can be seen in Figure 3b, which displays the results of the monitoring of the efflux of EtBr in the presence of DPM. The addition of PAβN and DPM (15.6 µg/mL and 7.8 µg/mL) could slow the decrease in EtBr fluorescence, suggesting their interference with EtBr efflux from the cells. The results of the dye efflux assays were consistent with the data from the dye accumulation assays, indicating that DPM could interfere with the efflux of H33342 and EtBr, therefore suggesting its EPI potential.

### 2.5. Drug Efflux Interference by DPM Monitored Using MALDI-TOF

Our modulation data indicated that DPM could reduce the IC_50_ of erythromycin, clarithromycin, and ciprofloxacin against Kam3-AcrB, and the dye accumulation/efflux data showed that DPM could increase H33342 and EtBr accumulation and reduce the dye efflux in Kam3-AcrB, possibly by interfering with the efflux pump. This suggests that DPM possesses EPI activity.

**Figure 4:**
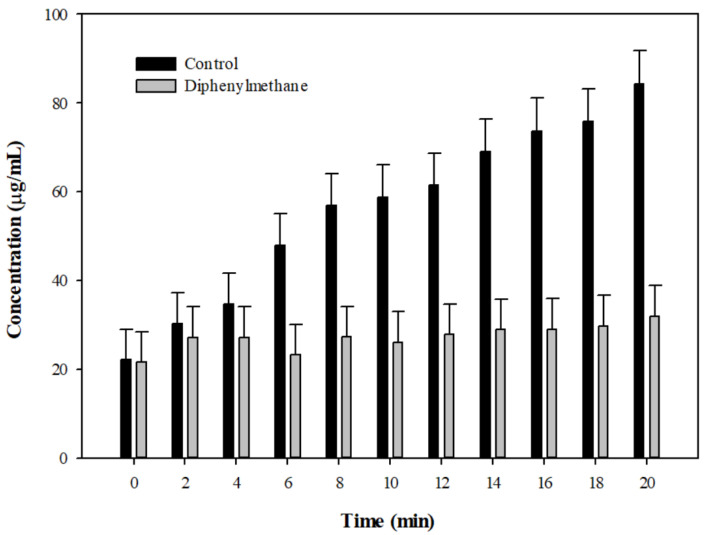
The erythromycin efflux activity of *E*. *coli* Kam3-AcrB was detected by using MALDI-TOF MS in the presence of diphenyl methane. The intensity was plotted at the main peak at *m*/*z* 738.35 of erythromycin and the detection period was 20 min. Values are expressed as mean ± SD (*n* = 3).

As described in this section, the drug transport was further investigated using MALDI-TOF MS to measure intracellular erythromycin in the presence of DPM as a function of time. We aimed to monitor the drug efflux instead of the dye efflux in the presence of DPM, providing direct evidence that DPM could interfere with antibiotic translocation by the efflux pump. The mass spectrum of erythromycin is shown in Supplementary Figure S2 and exhibits its main peak at *m*/*z* 738.847. As shown in Figure 4, the efflux of erythromycin by AcrB was measured by monitoring the intensity changes of extracellular erythromycin over time. The erythromycin concentration increased from 22.09 ± 6.90 µg/mL (t = 0 min) to 84.24 ± 7.5 µg/mL at 20 min in the extracellular space from Kam3-AcrB in the absence of DPM (Control), indicating the efflux of erythromycin by the energized *E. coli* Kam3-AcrB. Intriguingly, compared with the control, the extracellular erythromycin concentration of the Kam3-AcrB cells in the presence of DPM exhibited a significantly slower increase in erythromycin, with a final concentration of 31.87 ± 6.99 μg/mL (t = 20 min). When the Kam3-AcrB was energized by glucose in the absence of DPM, the intracellular erythromycin was transported out, and an increase in extracellular erythromycin was observed over time. The addition of putative EPI DPM could interfere with AcrB efflux in the *E. coli* Kam3-AcrB, and a minimum increase in extracellular erythromycin was observed. Our MS data indicated that DPM reduces erythromycin’s efflux by the energized *E. coli* Kam3-AcrB. The requirement for extensive hydrophobic interactions with RND transporters resulted in EPIs characterized either by insoluble or amphiphilic molecules [39]. For example, the crystal structures of D13-9001 bound to AcrB and MexB demonstrated binding to a unique site near the substrate’s deep binding pocket in the periplasmic domain, known as the hydrophobic trap [17].

Currently, the number of studies involving the use of mass spectrometry to monitor drug efflux by efflux pump is limited. High-performance LC–electrospray ionization–MS [40] and MALDI-TOF [3] have been reported to determine the intracellular and extracellular concentrations of drugs, respectively, for the direct detection of drug efflux. The other advantage of using Mass spectrometry to monitor drug efflux is to avoid any possible fluorescence interference from added EPIs, such as flavonoid quercetin, in dye accumulation/efflux experiments [41].

### 2.6. The Effect of DPM on Membrane Permeability and Post-Antibiotic Effect

**Figure 5:**
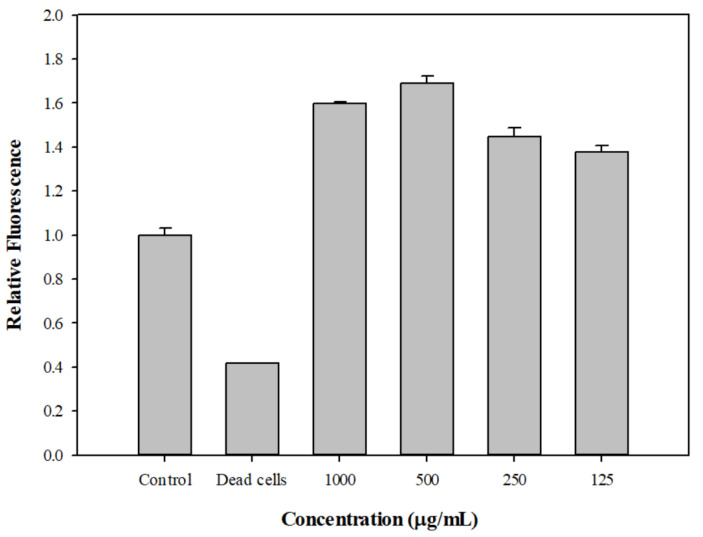
Effect of diphenyl methane on the membrane permeabilization of *E. coli* Kam3-AcrB. The membrane permeability was determined by using fluorescence dyes SYTO9 and propidium iodide, and the fluorescence was recorded at an Ex of 470 nm and an Em of 540 nm. Data are expressed as mean ± SD (*n* = 3).

The membrane comprises the major boundary outlining the cell cytoplasm, and the transmembrane electrochemical gradient powers many vital cellular functions. The maintenance of plasma membrane integrity is essential for normal cell viability and function. An increase in permeability could cause the dissipation of the proton motive force and the impairment of intracellular pH homeostasis. The effect of DPM on membrane permeability of Kam3-AcrB was determined by treating Kam3-AcrB with DPM in the presence of SYTO9 and propidium iodide. SYTO9 can penetrate cell membranes and exhibit fluorescence (green) upon DNA binding, whereas propidium iodide features a greater binding affinity to DNA than SYTO9, but cannot penetrate cell membranes. As shown in Figure 5, the fluorescence of SYTO9 from dead cells (heat inactivation) was significantly reduced. This was because the membrane permeability of the bacterial cells was disrupted upon heat treatment, and propidium iodide could penetrate the cell membranes to compete with SYTO9 for DNA binding, leading to reduced fluorescence from SYTO9. By contrast, the addition of DPM at tested concentrations did not reduce the SYTO9 fluorescence, suggesting that DPM does not increase cell membrane permeability. Ideal EPIs should not dissipate the energy source of the pump by disrupting the cytoplasmic membrane potential [42]. An effective EPI to inhibit the efflux pump by destabilizing the bacterial cell membrane and interfering with the energy might limit its use in clinical treatment due to its cell toxicity. For example, the protonophore CCCP is an effective EPI that can inhibit efflux pumps, such as *E. coli* AcrB [3], by creating membrane destabilization and interfering with proton motive force, but it has limited clinical use due to its cellular toxicity.

The post-antibiotic effect (PAE) refers to the continued suppression of bacterial growth after exposure to an antimicrobial agent [43], and it can be evaluated by measuring the time of no growth of the target organism after the removal of an antibiotic. As shown in Supplementary Table S1, the PAE values of erythromycin at IC_50_ and 2 × IC_50_ for Kam3-AcrB were 0.27 ± 0.02 and 0.30 ± 0.02 h, respectively. The combination of DPM with IC_50_ or 2 × IC_50_ erythromycin resulted in PAE values of 0.30 ± 0.00 and 0.27 ± 0.03, respectively. The PAE values of clarithromycin at IC_50_ and 2 × IC_50_ for Kam3-AcrB were 0.23 ± 0.06 and 0.27 ± 0.02, respectively, and the combined use of DPM plus IC_50_ or 2 × IC_50_ clarithromycin resulted in PAE values of 0.30 ± 0.00 and 0.27 ± 0.03, respectively. These data indicate that the addition of DPM does not seem to exert a significant effect on the PAE of erythromycin and clarithromycin. Tambat et al. [44] indicated that an indole of microbial origin, the 2-(2-aminophenyl) indole, could increase the PAE of ciprofloxacin against gram-positive bacterium *S. aureus* expressing efflux pump NorA by 1.1–1.9 h. For example, the combination of ciprofloxacin (8 μg/mL) and EPI 2-(2-aminophenyl) indoles (32 μg/mL) could extend the PAE for the *S. aureus* from 1.43 h (8 μg/mL ciprofloxacin alone) to 3.33 h. So far, few studies have reported that EPIs could extend the PAE of antibiotics against gram-negative bacteria. 

### 2.7. Cytotoxicity Test of DPM

**Figure 6:**
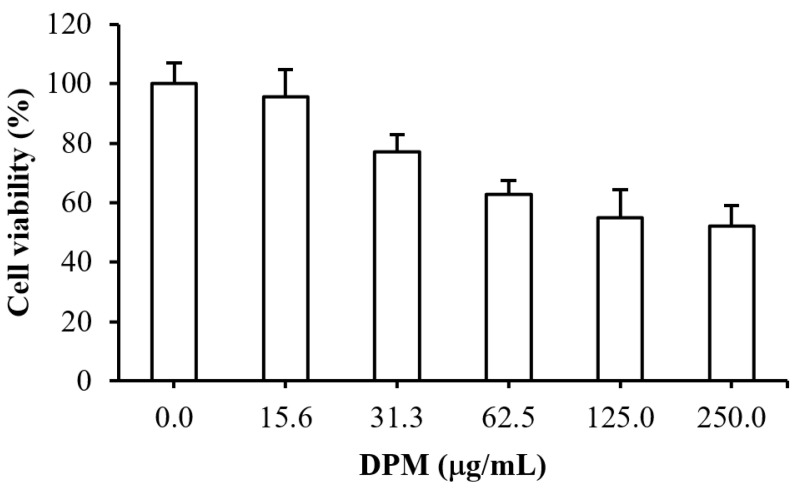
Cytotoxic effect of diphenylmethane on human hepatic HepG2 cells. DPM, diphenylmethane. Data are expressed as mean ± SD (*n* = 3).

The cytotoxicity test for the putative EPI DPM was performed by monitoring the viability of human hepatic HepG2 cells in the presence of various concentrations of DPM. As indicated in Figure 6, the cell viability for DPM at 15.6 µg/mL was 95.6% ± 4.0%, and the cell viability gradually decreased as the DPM concentration increased, with an IC_50_ > 250 μg/mL. Our previous data indicated that DPM at a concentration lower than 250 μg/mL could potentiate antibiotic activity, possibly by interfering with the drug efflux of Kam3-AcrB.

Alkaloid EPI piperine at 12.5 μg/mL (0.04 mM) reduces the MIC of ciprofloxacin twofold [19], and Ji, et al. [45] indicated that piperine at 32 μg/mL could reduce the MIC of ethidium bromide twofold against *Mycobacterium smegmatis*. Additionally, Jafri, et al. [46] observed that the viability of human Hela cells incubated with 0.050 mM, 0.1 mM, and 0.2 mM piperine was reduced by 30.1%, 50.73%, and 66.46%, compared with untreated cells. The modulation data indicated that the organic compound DPM could potentiate antibiotic clarithromycin, erythromycin, and ciprofloxacin against *E. coli* Kam3-AcrB at concentrations of 7.81 μg/mL (0.046 mM), 125 μg/mL, and 7.81 μg/mL, and the cytotoxicity data showed that the IC_50_ of DPM was determined to be >250 μg/mL (>1.48 mM) in human HepG2 cells, indicating that DPM could revive the antibiotic activities at sub-IC_50_ doses, possibly by interfering with the drug efflux of *E. coli* Kam3-AcrB.

## 3. Materials and Methods

### 3.1. Bacterial Strains, Constructs, Media, and Chemicals

An *acrB* deletion *E*. *coli* strain Kam3 (DE3) [47] and the construct *E. coli* Kam3 (DE3) harboring the pSYC plasmid encoding *acrB* were used for drug susceptibility, modulation, the dye/drug accumulation assays, the dye efflux assay, the membrane permeability assay, and the post-antibiotic effect evaluation. The bacteria were grown in Luria–Bertani broth (LB) and Mueller–Hinton broth (MH broth) for the cultivation and broth microdilution experiments. The erythromycin, clarithromycin, ciprofloxacin, tetracycline and DPM were purchased from Sigma-Aldrich (St. Louis, MO, USA). The DPM stock was prepared by dissolving DPM in 95% ethanol, and it was diluted 100-fold in a PBS buffer and media for the experiments in this study.

### 3.2. Identification of the Seaweed Compounds by Using GC-MS

The identification of DPM in *Gracilaria* sp. alcoholic extracts was accomplished by using a gas chromatography tandem mass spectrometer (GC-MS/MS, PolarisQ Ion Trap GC–MS/MS System, Thermo Scientific, Waltham, MA, USA). The Equity^®^−5 capillary column (length 30 m, inner diameter 0.25 mm, film thickness 0.25 μm) was used for separation in the gas chromatography, with helium (He) as the mobile phase and a flow rate of 1 mL/min. The sample was injected in a 10:1 split mode, with an injection temperature of 280 °C. The initial temperature during the separation was 50 °C for 2 min; the temperature was increased to 250 °C at a rate of 15 °C/min and maintained for 2 min. The mass spectrometry analysis used Electron Impact fragmentation, with an ion scanning range of 100–1000 *m*/*z*.

### 3.3. IC_50_ and Modulation Tests

The IC_50_ experiments were carried out as previously described, with some modifications [48]. The IC_50_ of the antibiotics erythromycin, clarithromycin, ciprofloxacin, and tetracycline against the drug-resistant *E. coli* strains were determined by using microdilution methods. The IC_50_ and modulation assays were performed using sterile 96-well microtiter plates containing the tested antibiotics and DPM in twofold serial concentrations. A serial dilution of the tested drugs in MH medium was performed from row A to G in a 96 well plate, and a serial dilution of DPM was performed from column 1 to 10. A solution of 20 μL containing various concentrations of antibiotics and DPM in each well was added with 180 μL of *E. coli* Kam3/ pSYC-*acrB* cells (5 Log CFU/mL), and the OD600 was checked after 12 h of incubation at 37 °C. The modulation factors for each antibiotic, along with their EPI concentrations, were recorded.

### 3.4. Time-Kill Assays

The time-kill experiments were performed according to the method described by Felicetti, et al. [49], with some modifications. Conical flasks with a volume of 50 mL, containing 20 mL *E. coli* Kam3/pSYC-*acrB* cells (5 Log CFU/mL), were added with erythromycin alone (250 μg/mL), DPM (125 and 250 μg/mL) or erythromycin and DPM, and the cell counts were monitored every 3 h over a period of 18 h by using plate counts. Each analysis was performed in triplicate.

### 3.5. Dye Accumulation Assay

The H33342 and EtBr dye accumulation assays were performed according to the method described in a previous report [50], with some modifications. A single colony of *E. coli* Kam3/pSYC-*acrB* was inoculated and cultivated in LB broth until an OD600 of approximately 0.8 was reached. The bacterial cells were centrifuged, washed twice, and resuspended in phosphate-buffered saline (PBS) (10 mM Na_2_HPO_4_, 1.8 mM KH_2_PO_4_, 137 mM NaCl, 2.7 mM KCl, 1 mM CaCl_2_, and 0.5 mM MgCl_2_ at pH 7.4) to an OD600 of approximately 0.5–0.6. A cell suspension of 150 μL was added with the filter-sterilized glucose (final conc 25 mM), the dyes (H33342 at 1 μM and EtBr at 2 μM), and various concentrations of DPM in a 96 well plate and the fluorescence were measured for 38 min (H33342, Ex: 360 nm, Em: 460 nm; EtBr, Ex: 520 nm, Em: 600 nm).

### 3.6. Dye Efflux Assay

The H33342 and EtBr dye accumulation assays were performed according to the method described in a previous report [44], with some modifications. The *E. coli* Kam3/pSYC-*acrB* solution for the dye efflux assay was prepared as described in Section 3.5. The bacterial solution was left for 9 h, and incubated with H33342 or EtBr (final concentration of 3 μM) for another 30 min. The *E. coli* cells were centrifuged, washed twice, and re-suspended in a PBS buffer to an OD600 of approximately 0.5–0.6. A cell suspension of 150 μL was added with the filter-sterilized glucose (final conc 25 mM), the dyes (H33342 at 1 μM and EtBr at 2 μM), and various concentrations of DPM in a 96 well plate, and the fluorescence was measured for 38 min (H33342, Ex: 360 nm, Em: 460 nm; EtBr, Ex: 520 nm, Em: 600 nm).

### 3.7. Monitoring Drug Efflux Using MALDI-TOF Mass Spectrometry

To monitor the erythromycin efflux in the presence of DPM by using MALDI-TOF, mass spectrometry was performed according to Lu et al. [3], with some modifications. A single colony of *E. coli* Kam3/pSYC-*acrB* was inoculated and cultivated in MH broth until an OD600 of approximately 1 was reached. The bacterial cells were centrifuged, washed twice, and resuspended in 10 mM ammonium bicarbonate solution to an OD600 of approximately 0.5–0.6. The bacterial solution was left at RT for 30 min and erythromycin, filter-sterilized glucose, and DPM were added subsequently. The samples were taken every two min for a total of 20 min. The samples were centrifuged at 6000× *g* for 1 min to obtain the supernatant, which was mixed with DHB matrix in a ratio of 1:1 and crystallized for MALDI-TOF MS analysis. The data acquisition was performed automatically (in random walk mode) in steps of 15 shots, for a total of 3000 shots per sample. FlexAnalysis (version 3.0, Bruker Daltonics, Billerica, MA, USA) software was used for the data analysis.

### 3.8. Membrane Permeability Assay

The membrane permeability assay was performed according to Machad, et al. [51], with some modifications. A single colony of *E. coli* Kam3/pSYC-*acrB* was inoculated and cultivated in MH broth until an OD600 of approximately 1 was reached. The bacterial cells were centrifuged and resuspended in PBS to an OD600 of approximately 0.5–0.6. 100 μL of bacterial solution and 100 μL of dye mixture (SYTO 9 to propidium iodide ratio equals 1:1) were mixed and added into a black 96 well plate, and the mixtures were incubated for 15 min at RT in the dark before the measurement of fluorescence (Ex: 470 nm, Em: 540 nm).

### 3.9. Post-Antibiotic Effect Assay

This experiment was performed according to the method described in a previous report [44]. *E. coli* Kam3/pSYC-*acrB* was cultivated at 37 °C and 150 rpm to reach an OD600 of approximate 0.5–0.6. The bacterial cultures were divided into three groups: control, the cultures to which antibiotics (IC_50_ and 2 × IC_50_ concentrations) were added, and the cultures to which antibiotics (IC_50_ and 2 × IC_50_ concentrations) + DPM (125 μg/mL) were added. The bacterial culture was cultivated for another 2 h after the antibiotics or antibiotics/DPM were added. The bacterial cultures were inoculated into new LB broth at a thousand-fold dilution, and the cell counts (CFU/mL) were measured every hour by using plate counts until tenfold of the cell counts were reached. Subsequently, the time required for the bacterial counts to increase by 10 folds at each group can be determined.

### 3.10. Cell Toxicity Assays

A human hepatic HepG2 cell line was maintained according to the instructions of the Bioresource Collection and Research Center (BCRC; Hsinchu City, Taiwan), and supplemented with 50 units/mL penicillin G and 50 µg/mL streptomycin sulfate. All of the reagents for the cell culture were obtained from Gibco/Thermo Fisher Scientific Inc. (Bethesda, MD, USA). The cytotoxicity of DPM on HepG2 cells was estimated through a trypan blue exclusion assay. Approximately 4 × 10^4^ cells were seeded in a 12-well culture plate, and then incubated overnight. The DPM was solved in ethanol, and then added to the culture medium at final concentrations of 0, 15.6, 31.3, 62.5, 125, and 250 µg/mL. The cells were incubated for 24 h before the cell viability assay.

### 3.11. Statistical Analysis

The data were analyzed statistically by using SPSS version 12 (SPSS Inc.Chicago, IL, USA) and presented as means ± standard deviation. One-way analysis of variance (ANOVA) was used to determine statistical differences between the sample means, with the level of significance set at *p* < 0.05, and multiple comparisons of means were obtained through a Tukey test.

## 4. Conclusions

Gram-negative pathogen *E. coli* is one of the most troublesome clinical bacterial species, and very few EPIs for gram-negative bacteria have been reported. The data from this study indicated that DPM could potentiate antibiotic activity for drug-resistant *E. coli* by reducing the efflux pump efficiency. Additionally, the DPM did not seem to damage the *E. coli* cell membrane and showed mild human-cell toxicity. The IC_50_ of DPM for human HepG2 cells was determined, and it was higher than the effective doses for the antibiotic potentiation in this study. To the best of our knowledge, this is the first compound of seaweed origin to have been identified as exhibiting EPI activity. In conclusion, DPM could serve as a potential EPI against drug-resistant *E. coli*, and further pharmacokinetic and in vivo studies should be conducted in the future to confirm its clinical efficacy.

## Figures and Tables

**Table 1:** Modulation activity of diphenylmethane for the antibiotics against *E. coli* Kam3-AcrB.

Antibiotics	DPM Concentration(µg/mL)	IC_50_ (µg/mL)		Modulation Factor
		Alone	With DPM	
Clarithromycin	7.81	175	87.5	2
Erythromycin	125	125	62.5	2
Ciprofloxacin	7.81	0.06	0.03	2

DPM, Diphenyl methane.

## Supplementary Materials

The following are available online at https://www.mdpi.com/article/10.3390/antibiotics10111378/s1. Table S1: Post-antibiotic effect of DPM and antibiotics on *E. coli* Kam3 harboring pSYC-*acrB*. Figure S1: Chemical structure of diphenylmethane identified in red seaweed *Gracilaria* sp. by GC-MS. Figure S2: The mass spectrum of erythromycin.
