# Supplementary material for: Identified Seaweed Compound Diphenylmethane Serves as an Efflux Pump Inhibitor in Drug-Resistant Escherichia coli
Source: Antibiotics (Basel). 2021 Nov 10;10(11):1378. doi: 10.3390/antibiotics10111378 (PMC8614644; doi:10.3390/antibiotics10111378)

Supplementary Table S1. Post-antibiotic effect of DPM and antibiotics on *E. coli* Kam3 harboring pSYC-acrB.

Regimen	Mean PAE (h) ± SD	
	Erythromycin	Clarithromycin
IC_{50} Drug	0.27 ± 0.02	0.23 ± 0.06
IC_{50} Drug + DPM	0.30 ± 0.00	0.21 ± 0.01
2 × IC_{50} Drug	0.30 ± 0.02	0.27 ± 0.02
2 × IC_{50} Drug + DPM	0.27 ± 0.03	0.34 ± 0.03

IC_{50}, Erythromycin (125 μg/mL); Clarithromycin (175 μg/mL); DPM, diphenylmethane (125 μg/mL).

Supplementary Figure S1. Chemical structure of diphenylmethane identified in red seaweed *Gracilaria* sp. by GC-MS.

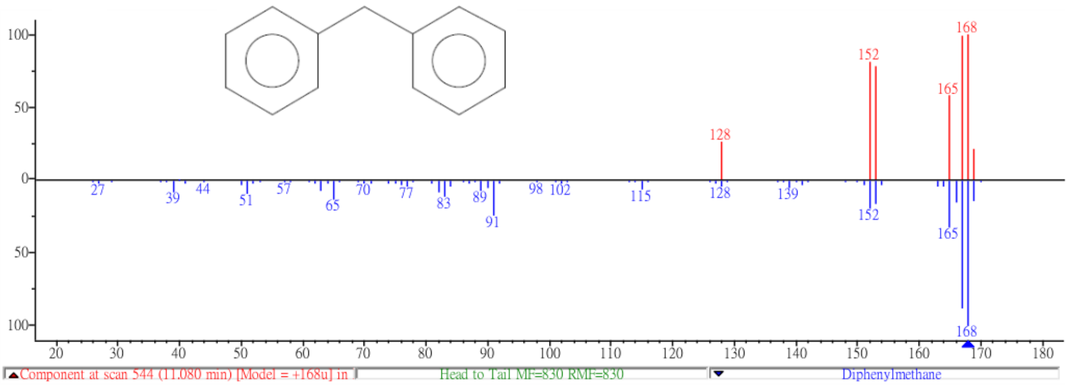

Supplementary Figure S2. The mass spectrum of erythromycin.

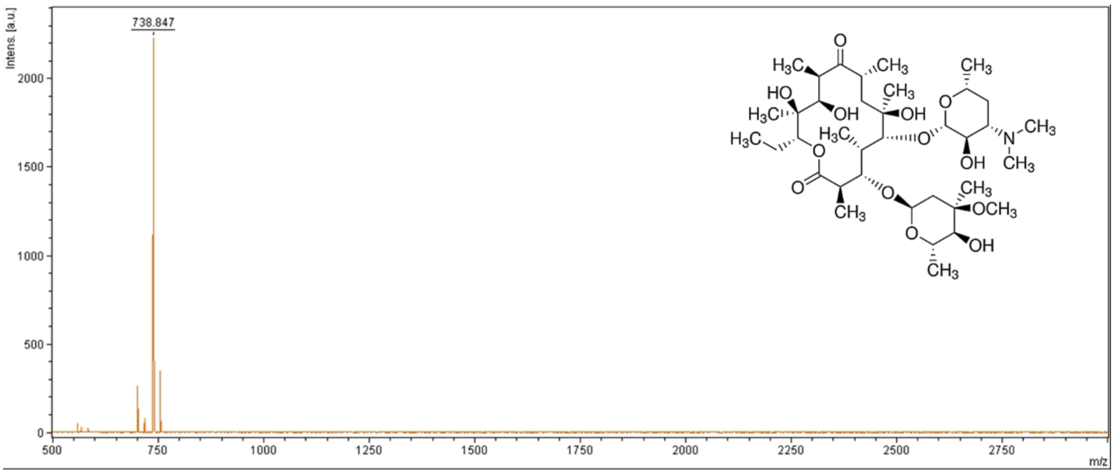

Supplement: Supplementary file 1 [file antibiotics-10-01378-s001.zip › antibiotics-1432593-supplementary.pdf]
